# Modeling foreign language majors’ adoption intention of Chinese GenAI tools: an extended TAM with perceived teacher attitude and peer influence

**DOI:** 10.3389/fpsyg.2026.1850558

**Published:** 2026-06-30

**Authors:** Mei Huang, Ling Huang

**Affiliations:** 1 School of Foreign Language and International Business, Guilin University of Aerospace Technology, Guilin, Guangxi, China; 2 Graduate Institute of Interpretation and Translation, Shanghai International Studies University, Shanghai, China; 3 Liberal Arts College, Hunan Normal University, Changsha, Hunan, China

**Keywords:** adoption intention, Chinese GenAI tools, foreign language majors, peer influence, perceived teacher attitude, technology acceptance model (TAM)

## Abstract

This study explores the key determinants shaping foreign language majors’ adoption intention toward Chinese generative artificial intelligence (GenAI) tools. Using an extended Technology Acceptance Model (TAM), it examines the underexplored roles of two critical social factors: perceived teacher attitude (PTA) and peer influence (PI). A quantitative methodology was adopted, utilizing an online questionnaire to collect data from 423 foreign language majors across multiple universities in China. The proposed theoretical model and hypothesized relationships were rigorously analyzed through structural equation modeling (SEM). The results identified PI and attitude as direct positive predictors of adoption intention. Notably, restrictive PI was not significantly associated with adoption intention, whereas supportive PTA showed a significant positive association. This research extends the TAM and implied that simple prohibitions or passive advocacy are insufficient to promote the effective application of AI. Educational stakeholders should take practical measures to go with the future trends in Human-Machine Collaboration.

## 1 Introduction

The rapid advancements in artificial intelligence (AI) are driving a wave of innovation in language education (Moussalli and Cardoso, 2020), providing personalized support that enhances proficiency, motivation, and autonomy (Liang et al., 2021). Specifically in computer-assisted language learning, AI significantly boosts interactivity, feedback timeliness, and resource integration (Liu and Ma, 2023). However, these advancements pose challenges for foreign language majors. Unlike general English learners who treat the language as a purely instrumental subject, undergraduate English programs aim to cultivate specialized language professionals, cross-cultural communicators, and literary researchers. Yet mainstream AI language tools can now efficiently handle routine translation, basic copywriting, grammar correction, and voice dialogue, which are core competencies traditionally mastered by foreign language majors. In certain industry contexts, AI can handle over 92% of routine language service tasks. This directly undermines the talent development positioning of traditional language programs and erodes students’ core professional competitiveness, plunging foreign language majors into anxiety about their academic development and uncertainty about their career prospects.

Meanwhile, foreign language teaching in Chinese universities remains largely entrenched in traditional models. Most institutions have yet to adjust their talent cultivation plans in response to the AI wave. Faculty AI literacy varies significantly, leaving educators unaware of students’ actual usage patterns, acceptance levels, and practical needs regarding AI language tools. Without targeted guidance strategies, students’ use of AI is largely limited to the superficial need to complete assignments, and they lack the awareness and ability to engage in deep collaboration with AI. This not only leads to a decline in students’ actual proficiency in traditional language skills but also reduces the “AI-assisted learning” approach to a mere formality.

As Moradi (2025) observed, shaped by their unique cultural, social, and educational pressures, English as a Foreign Language (EFL) learners in China have developed distinct patterns in their use of AI tools, making them a subject of significant research value. While foreign language majors in China have grown increasingly reliant on AI tools, their usage landscape differs markedly from the international norm. Unlike the prevalent focus in existing research on global models such as ChatGPT (Liu and Ma, 2023; Moradi, 2025), the Chinese environment is uniquely shaped by accessibility, cost, and data security considerations, which have limited the adoption of international tools (Ma, 2025). Instead, domestically developed GenAI applications, such as DeepSeek, Doubao, and ERNIE Bot, have gained prominence due to their free access, user-friendly design, faster processing speeds, and localized support (Wu and Zhang, 2024; Zhang and Yu, 2025). Nevertheless, despite their widespread use, few studies have systematically investigated the acceptance of these local tools, a gap this study aims to address by examining adoption intention among foreign language majors.

This study is grounded in local AI contexts and expands the application of the Technology Acceptance Model (TAM) in the field of professional foreign language education by introducing perceived teachers attitudes (PTA) and peer influence (PI) as external variables. The findings are expected to provide tiered decision-making references for higher education administrators, foreign language teachers, and English majors. They will help clarify the core factors influencing English majors’ acceptance of AI within the dimensions of the teaching environment and social interaction, thereby facilitating adjustments to talent development programs, the transformation of teachers’ roles, and the restructuring of students’ competencies. This study aims to offer practical solutions with both local applicability and broader value for AI-enabled foreign language education reform with Chinese characteristics.

## 2 Literature review

### 2.1 The technology acceptance model and its evolution

First proposed by Davis in 1989, the TAM was developed to account for and forecast individuals’ adoption of information technology (Hsu, 2022). Rooted in the Theory of Reasoned Action, this model centers specifically on technology acceptance, presenting a more concise theoretical framework via its simplified structural design (Lai and Lee, 2020).

The core constructs of TAM consist of two key variables: Perceived Usefulness (PU) and Perceived Ease of Use (PEU) (Davis, 1989). PU is defined as “the degree to which people believe that using a particular system would enhance their job performance,” while PEU refers to “the degree to which people believe that using the system would be free of effort” (Davis, 1989). These two variables are collectively associated with users’ attitude toward using (ATT) the technology and their behavioral intention (BI) to use it (Panopoulou et al., 2021). Given its parsimonious design and powerful explanatory strength, this model has established itself as a fundamental theoretical basis for research on technology acceptance in educational contexts (Feng et al., 2021).

TAM has evolved through multiple extensions. Many extended TAM studies in educational contexts incorporate student attitude and self-efficacy as key antecedents, forming a mediating path via system usage to predict learning system effectiveness (Hussein et al., 2024). Others incorporated variables such as social influence (SI) processes, significantly enhancing the model’s explanatory power regarding the social dimension of technology acceptance. This integration of social factors paved the way for subsequent research to explore more nuanced social dynamics in technology adoption. For instance, the e-Participation Acceptance Model integrates “SI” as one of its core constructs, further underscoring the importance of social factors in technology adoption (Panopoulou et al., 2021).

It is worth noting that most early extended TAM models conceptualize social influence as a general, unified construct, and rarely differentiate diverse sources of social influence. This oversimplification limits the model’s explanatory power in interactive and layered environments such as classrooms.

The recognition of SI makes TAM particularly pertinent to educational settings. Consequently, TAM and its extensions have been widely applied to examine teachers and students’ technology acceptance (Kohnke et al., 2023; Ma, 2025; Meyer et al., 2024; Wang and Wang, 2025). A comprehensive review of 71 studies confirmed the model’s efficacy in explaining behavioral intentions across diverse educational contexts (Granić and Marangunić, 2019), including the adoption of various digital tools from e-learning systems to AI-powered chatbots (Ma, 2025).

Despite abundant applications in general education, relevant studies seldom adjust the framework to fit the characteristics of AI-assisted language learning, nor do they distinguish the respective roles of teachers and peers within social influence. These gaps provide the starting point for the present study’s model design.

### 2.2 AI in language learning

The integration of AI-based systems in education has become a rapidly growing area of research. Scholarly interest increasingly focuses on understanding their acceptance, behavioral intention, and actual use by learners and educators (Li, 2023). With its capacity for personalized adaptation and contextually situated interaction, GenAI is increasingly becoming a key component in global strategies to enhance language acquisition effectiveness (Zheng et al., 2024).

The benefits of this integration are multifaceted. Research shows that GAI tools facilitate language skill development through instant feedback that mirrors natural social interaction (Meyer et al., 2024). This is exemplified by AI-driven chatbots, which significantly enhance proficiency in writing, grammar, and vocabulary, thereby boosting student motivation and involvement (Kohnke et al., 2023). Furthermore, AI technologies beyond GenAI also enhance critically: they facilitate language learning by processing oral input, generating spoken responses, and providing diagnostic assessments (Chen, 2022); adaptive learning systems tailor educational content recommendations to individual learners based on their competency and preferences (Liu and Ma, 2023).

Despite its significant benefits, AI introduction poses distinct challenges for language learners. AI can exhibit limitations in oral communication and semantic understanding, potentially leading to learner dissatisfaction and suboptimal communication outcomes (Chen, 2022). Furthermore, AI-driven systems may overemphasize technological intervention by weakening human interaction, thereby hindering the development of pragmatic language application skills (Kohnke et al., 2023). An overreliance on AI for language assessment has also been linked to increased anxiety among learners (Zhang, 2024).

### 2.3 The Chinese context: adoption of domestic GenAI tools in foreign language learning

China’s GenAI sector is experiencing vigorous growth, underpinned by a series of supportive government policies. The early *New Generation Artificial Intelligence Development Plan* that put forwards by the State Council in 2017 has recently been accelerated in the education sector by explicit mandates, such as those in the 2025 Opinions on Accelerating Educational Digitalization, to deepen the application of educational large language models. In response, leading Chinese companies like Baidu, ByteDance, and DeepSeek have developed significant products (e.g., ERNIE Bot, DouBao, DeepSeek), which now offer crucial support for personalized learning, teaching assistance, and public knowledge services.

The applications of domestically developed GenAI tools are primarily manifested in several key areas, including language learning assistance, writing support, intelligent tutoring, and speaking practice etc.

Studies on various Chinese generative AI tools highlight their diverse applications in language learning. An investigation into the DouBao chatbot revealed its efficacy in simulating authentic conversations and assisting with fundamental tasks like vocabulary explanation, grammar correction and text generation (Zhang and Yu, 2025). Similarly, research on ERNIE Bot identifies its dual role as a “virtual tutor” for personalized instruction and an “interactive practice partner” to prevent error fossilization (Wu and Zhang, 2024). Furthermore, research on DeepSeek confirms its significant positive association with EFL writing performance, particularly in micro-level competencies (Zhao et al., 2025).

Domestically developed generative AI tools, however, are not without their limitations in foreign language learning. For instance, DeepSeek demonstrates deficiencies in areas such as grammar error correction, spoken fluency feedback, listening comprehension assessment, and writing coherence evaluation (Zhao et al., 2025). Meanwhile, ERNIE Bot still faces challenges when processing complex linguistic patterns, such as metaphors in literary texts or cultural loaded expressions, which may compromise the accuracy of its teaching or interactive support (Wu and Zhang, 2024). The limitations of Doubao in language learning involve algorithmic bias, fostering over-reliance, and technical integration hurdles (Lin and Li, 2025).

Nevertheless, existing research exhibits a notable limitation by predominantly focusing on technical capabilities while overlooking the role of social factors on student adoption. However, SI is important for it may function distinctly within China’s collectivist culture (Zhao et al., 2021). Rooted in the norm of respecting teachers, educator attitudes are widely believed to shape behavioral intentions. Similarly, within collective university environments, peer influence, evidenced by reliance on friend recommendations (Ma, 2025), is particularly pronounced. On the other hand, most studies target non-specialist EFL learners, creating a scarcity of research on foreign language majors, a distinct population whose specialized curriculum and deeper engagement with language likely foster unique adoption patterns.

### 2.4 Extending the TAM: the key roles of perceived teacher attitude and peer influence

Venkatesh et al. (2003) conceptualizes SI as “the degree to which people perceive that important others believe they should use the new system.” This concept finds a more applicable context in the model of Unified Theory of Acceptance and Use of Technology 2 (UTAUT2) (Venkatesh et al., 2012), which contextualizes SI within voluntary settings.

Most prior TAM and UTAUT extensions in educational technology research adopt SI as a single holistic construct and rarely decompose it into different sources. This general treatment cannot fully capture the layered interpersonal interactions inside classrooms.

This effect is especially pronounced in collectivist educational settings across Asia, where the uptake of technological tools is frequently driven in large part by the attitudes of teachers and fellow students (Habibi et al., 2023). Nevertheless, few existing studies on AI-assisted language learning have separately examined the impacts of teacher and student groups, leading to incomplete understanding of social influence mechanisms. Therefore, to precisely delineate the mechanisms of SI, this study operationalizes it through two specific variables: PTA and PI.

PTA can be defined as a student’s inference of whether their instructors positively or negatively view the academic use of GenAI tools. This perception, which may be formed through explicit instructions, implicit cues, or anticipated evaluations, constitutes a form of top-down SI that shapes the student’s own adoption intentions.

PI refers to the social pressure and behavioral norms emanating from a student’s fellow students regarding the use of GenAI tools. It encompasses observational learning, social comparisons, direct recommendations, and the need for conformity within the academic cohort, representing a horizontal channel of social influence.

Teachers’ attitudes and peer interactions are critical social factors shaping foreign language learners’ motivation and technology adoption behavior (Hatamleh et al., 2025). In educational settings, teachers and peers constitute the primary sources of social influence that shape students’ attitudes and behaviors. While other constructs such as trust, perceived risk, AI anxiety, and self-efficacy may also influence technology adoption, they were beyond the scope of this study, which focused specifically on social influence mechanisms. Future research should extend the model by incorporating these additional variables to further enhance its explanatory power.

### 2.5 Research gaps and hypothetical models

The existing literature reveals three primary gaps. First, the application of the TAM to domestic AI tools is limited. Second, the adoption dynamics of these tools among Chinese foreign language majors remain underexplored. Finally, the specific mechanisms linking PTA and PI with core TAM variables are not yet clearly understood.

**Figure 1:**
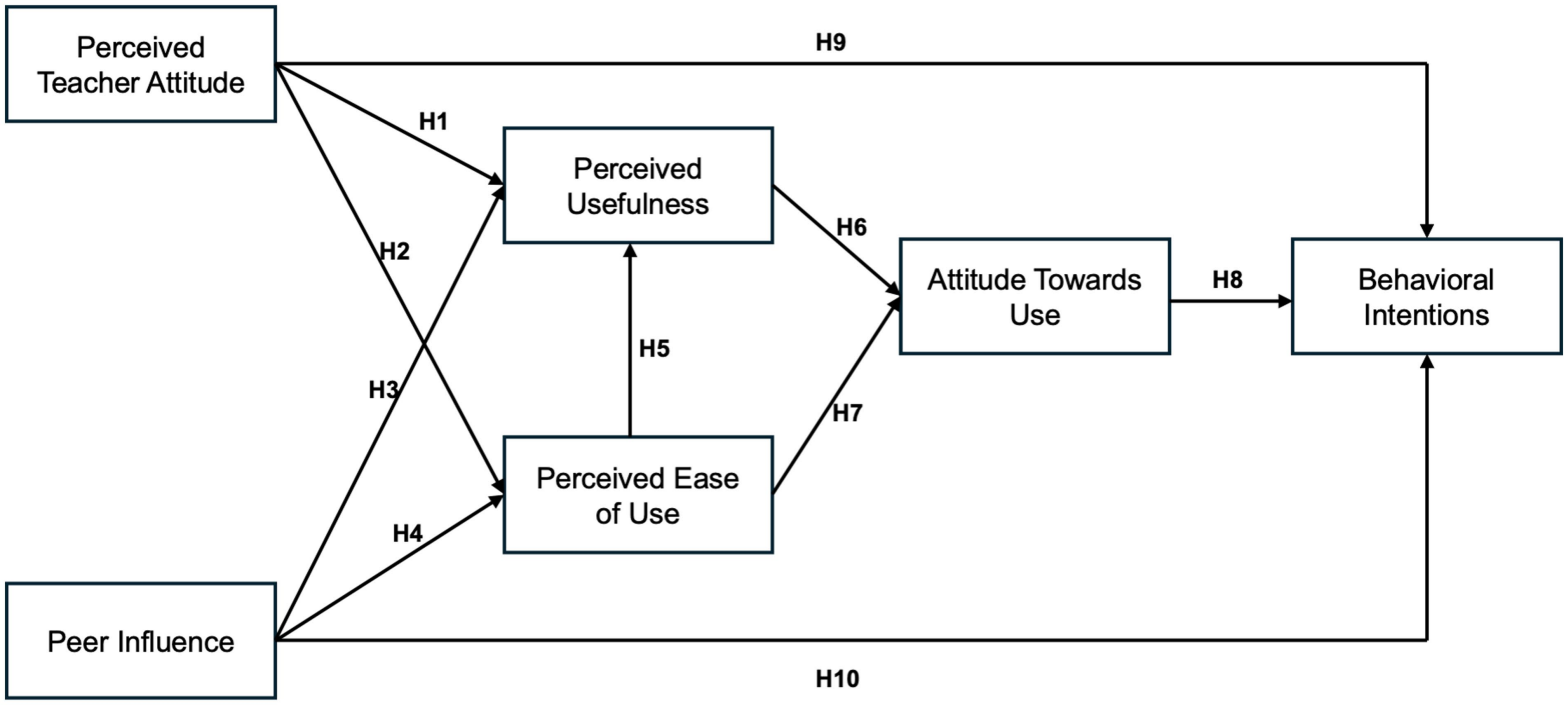
Hypothetical structural model predicting foreign language learners’ behavioral intentions to adopt Chinese generative AI language learning tools. H1–H10 label all proposed direct predictive paths. Exogenous variables include Perceived Teacher Attitude and Peer Influence; Perceived Usefulness and Perceived Ease of Use act as TAM mediating constructs, and Attitude Towards Use serves as the proximal predictor of Behavioral Intentions.

Based on the relevant literature and research gaps, a conceptual model was developed integrating PTA, PI, PU PEU, ATT, and BI. The hypothesized model (Figure 1) gives rise to the following hypotheses, which are summarized in Table 1.

**Table 1:** Summary of hypotheses in the hypothesized model.

Hypothesis	Content
Hypothesis 1	Perceived teacher attitude towards Chinese GenAI tools positively predicts foreign language majors perceived usefulness of these tools.
Hypothesis 2	Perceived teacher attitude towards Chinese GenAI tools positively predicts foreign language majors perceived ease of use of these tools.
Hypothesis 3	Peer influence positively predicts the perceived usefulness of Chinese GenAI tools.
Hypothesis 4	Peer Influence positively predicts the perceived ease of use of Chinese GenAI tools.
Hypothesis 5	Perceived ease of use of Chinese GenAI tools positively predicts the perceived usefulness among foreign language majors.
Hypothesis 6	The perceived usefulness of Chinese GenAI tools positively predicts foreign language majors’ attitude towards using them.
Hypothesis 7	The perceived ease of use of Chinese GenAI tools positively predicts foreign language majors’ attitude towards using them.
Hypothesis 8	Foreign language majors’ attitude towards Chinese GenAI tools positively predicts their behavioral intentions to use them.
Hypothesis 9	Perceived teacher attitude toward Chinese GenAI tools positively predicts foreign language majors’ adoption intention.
Hypothesis 10	Peer influence regarding Chinese GenAI tools positively predicts foreign language majors’ adoption intention.

## 3 Method

### 3.1 Participants

In this study, the 423 participants were foreign language majors from 6 undergraduate universities, located in Guangxi Zhuang Autonomous Region and Hunan Province, two bordering provincial level administrative regions in southern China.

The sample comprised 72 male students (17.0%) and 351 female students (83.0%), a distribution that aligns with the notable gender imbalance typically found in foreign language programs across Chinese higher education. In terms of grade level, 171 respondents (40.4%) were freshmen and sophomores, while 252 (59.6%) were juniors and seniors. All participants were specializing in language related disciplines.

The questionnaire further investigated participants’ familiarity with and usage frequency of Chinese GenAI tools (e.g., DeepSeek, Doubao, ERNIE Bot). The results revealed that most of the sample was moderately familiar with these tools. Moreover, a high percentage of participants reported using them occasionally or frequently for foreign language learning. A summary of the descriptive statistics for these core variables can be found in Table 2.

**Table 2:** Participants’ demographic information (*N* = 423).

Demographic profile	Category	Number	Percent
Gender	Male	72	17
	Female	351	83
Grade	Lower	171	40.4
	Higher	252	59.6
Major	English	157	37.1
	Translation	77	18.2
	Business English	82	19.4
	Other (Japanese, Thai, Spanish, Korean, French, Russian, etc.)	107	25.3
Familiarity with Chinese GenAI tools (e.g., DeepSeek, Doubao, ERNIE Bot)	Slightly familiar	9	2.1
	Somewhat familiar	140	33.1
	Moderately familiar	220	52
	Very familiar	54	12.8
Usage frequency of Chinese GenAI tools for foreign language learning	Rarely	17	4
	Occasionally	189	44.7
	Frequently	194	45.9
	Always	23	5.4

### 3.2 Instruments

Data were collected using a Chinese online survey platform called Chaoxing. The instrument comprised three sections (see Appendix for the full questionnaire). The first section gathered sociodemographic details (e.g., gender, major, academic year) and assessed familiarity and usage frequency of Chinese GenAI tools. The second section measured the core TAM constructs (PU, PEU, ATT, and BI) through 13 items, adapted from the established scales of Davis (1989) and Venkatesh et al. (2003). The third section addressed the two external variables, PTA and PI, with four items for each construct, for a total of eight items; the PTA items are drawn from the established scale by Venkatesh et al. (2012) while the PI items are adapted from the classic scale by Ajzen (1991). All items in sections two and three employed a five-point Likert scale (1 = Strongly Disagree to 5 = Strongly Agree). All measurement items were originally developed in English. To ensure linguistic accuracy and cultural appropriateness for Chinese foreign language majors, standard scale adaptation procedures were followed. First, all items were translated into Chinese and then back translated into English by two independent bilingual researchers to guarantee semantic equivalence. Second, two experts in educational technology and applied linguistics reviewed the translated scale for content validity, wording clarity, and cultural consistency. Third, a pilot test involving 50 undergraduate foreign language students was conducted. Minor linguistic revisions were made based on pilot feedback to adapt the items to the local educational context.

### 3.3 Data collection and analysis

A total of 455 complete questionnaires were received at the initial stage. Subsequent data cleaning was conducted to remove samples with excessively short response times, overly consistent answers, and contradictory responses. The final sample consisted of 423 valid responses, accounting for 92.9% of the total questionnaires distributed.

All statistical analyses were conducted via SPSS 29.0 and RStudio 2025.05.1. To begin, initial data screening and descriptive analyses were carried out in SPSS 29.0. For reliability evaluation, the internal consistency of the measurement scales was examined via Cronbach’s alpha coefficient, with a widely accepted cutoff of *α* > 0.7 applied to both the overall scale and each individual construct (Fornell and Larcker, 1981). Principal component analysis was employed for factor extraction, and exploratory factor analysis (EFA) was conducted using maximum variance rotation. This approach was chosen for data reduction purposes. Components with eigenvalues greater than 1 were retained. To refine the scale, items were removed if their component loadings were below 0.5 or if they exhibited significant cross-loadings (Hair et al., 2010). Concurrently, descriptive statistics were calculated for all variables, and data normality was tested using skewness (absolute value < 3) and kurtosis (absolute value < 8) (Kock and Moqbel, 2016) to satisfy the prerequisites for subsequent modeling.

Subsequently, Confirmatory Factor Analysis (CFA) and SEM were conducted in RStudio. The maximum likelihood estimation method was employed. Factor loadings were estimated using the lavaan package. Convergent validity was assessed using the Average Variance Extracted (AVE > 0.5) and Composite Reliability (CR > 0.7), while discriminant validity was verified via the Fornell–Larcker criterion (Fornell and Larcker, 1981). Once the fit of the measurement model was verified, structural model was constructed to investigate how external variables relate to BI via the mediating pathway of the core TAM constructs. The significance of indirect effects was examined via a bias-corrected percentile bootstrap approach based on 5,000 resamples. Statistical significance was established when the 95% confidence interval (CI) excluded zero (Hayes, 2013).

Model fit was evaluated using a combination of widely accepted indices and criteria (Hair Jr et al., 2014). The following thresholds were applied for acceptable model fit: a chi-square to degrees of freedom ratio (*χ*^2^/df) below 5.0, a Root Mean Square Error of Approximation (RMSEA) less than 0.10, a Comparative Fit Index (CFI) exceeding 0.90, and a Tucker-Lewis Index (TLI) above 0.90.

To assess the potential threat of common method bias, we conducted Harman’s single-factor test (Podsakoff et al., 2003). All measurement items were entered into an unrotated principal component factor analysis. The results revealed that the first unrotated factor accounted for 28.20% of the total variance, which is well below the recommended threshold of 50%. This suggests that common method bias is unlikely to be a serious concern in this study.

Additionally, gender, familiarity with GenAI translation tools, and frequency of GenAI use were incorporated as control variables in the structural model to account for individual differences that might influence adoption intention.

## 4 Results

### 4.1 Reliability and validity

The questionnaire exhibited strong internal consistency, evidenced by an overall Cronbach’s *α* of 0.862 and construct level values were between 0.766 to 0.842, thereby exceeding the recommended 0.70 threshold (Nunnally and Bernstein, 1994). Furthermore, the data demonstrated no significant deviations from univariate normality, with skewness (−1.281 to 0.586) and kurtosis (−0.133 to 0.359) values all falling within the conservative thresholds of ±3 and ±8, respectively (see Table 3). Multivariate normality was assessed using standardized residuals. The results showed that the data violated the multivariate normality assumption. Given that the data were measured on a five-point Likert scale, the robust maximum likelihood (MLR) estimator was employed in lavaan. This estimator provides corrected standard errors and test statistics under non-normality and is widely recommended for ordinal Likert-type data in SEM analyses.

**Table 3:** Reliability, validity, descriptive statistics.

Variables	Cronbach’s *α*	M	SD	Skewness	Kurtosis	AVE	CR
PTA	0.766	3.835	0.719	0.056	−0.674	0.465	0.776
Supportive PTA	Single item	4.00	0.77	−0.56	0.31	Single item	Single item
Restrictive PTA	0.73	3.78	—	−0.54	−0.11	0.482	0.736
PI	0.82	3.845	0.646	0.24	−0.348	0.537	0.822
PU	0.802	3.833	0.671	−0.117	−0.095	0.523	0.814
PEU	0.824	3.753	0.864	−0.133	−1.281	0.613	0.826
ATT	0.842	3.991	0.705	−0.013	−0.85	0.645	0.845
BI	0.83	3.846	0.618	−0.107	0.586	0.626	0.834

The original PTA construct was further separated into supportive PTA (single indicator: PTA3) and restrictive PTA (PTA1, PTA2, PTA4) for supplementary empirical testing. Values of Cronbach’s α, AVE and CR are unavailable for single-item construct. Supportive PTA was measured with a single item (PTA3), so reliability and convergent validity indices cannot be calculated. PTA, perceived teacher attitude; PI, peer influence; PU, perceived usefulness; PEU, perceived ease of use; ATT, attitude toward use; BI, behavioral intention; M, mean; SD, standard deviation.

Validity and reliability were assessed using CFA. As shown in Table 4, the standardized factor loadings for each measurement item range from 0.66 to 0.84, with all loadings exceeding the threshold of 0.6 for good convergence validity. Furthermore, all items were found to be statistically significant at the *p* < 0.001 level, clearly indicating that the measurement model in this study demonstrates good convergence validity.

**Table 4:** Results of load standardization factors for the measurement model.

Construct	Item	Standardized factor loading	*p*-value
PTA	PTA1	0.73	<0.001
	PTA2	0.67	<0.001
	PTA3	0.67	<0.001
	PTA4	0.66	<0.001
PI	PI1	0.70	<0.001
	PI2	0.75	<0.001
	PI3	0.79	<0.001
	PI4	0.68	<0.001
PU	PU1	0.72	<0.001
	PU2	0.75	<0.001
	PU3	0.66	<0.001
	PU4	0.77	<0.001
PEU	PEU1	0.77	<0.001
	PEU2	0.84	<0.001
	PEU3	0.74	<0.001
ATT	ATT1	0.82	<0.001
	ATT2	0.78	<0.001
	ATT3	0.81	<0.001
BI	BI1	0.74	<0.001
	BI2	0.83	<0.001
	BI3	0.80	<0.001

All indicator loadings achieved statistical significance at the *p* < 0.001 threshold, with the benchmark for factor loadings is: ≥ 0.6 is considered acceptable. See Table 3 for abbreviation definitions.

Convergent validity was evaluated using AVE and CR (see Table 3). To verify the two-factor structure of PTA, a domain-specific two-dimensional CFA was performed. The two-dimensional CFA model showed excellent fit to the data: *χ*^2^(172) = 287.87, CFI = 0.967, TLI = 0.960, RMSEA = 0.040, 90% CI [0.032, 0.048]), SRMR = 0.052, all meeting recommended thresholds (Hu and Bentler, 1999; Kline, 2015), confirming the feasibility of splitting PTA into supportive PTA and restrictive PTA. Supportive PTA was measured by one item reflecting teachers’ acknowledgment of GenAI value (PTA3), and restrictive PTA was measured by three items reflecting boundary setting, prohibition, and criticism (PTA1, PTA 2, PTA5).

For restrictive PTA, AVE was slightly below at 0.482. However, convergent validity is still considered adequate if the CR is sufficiently high even with a lower AVE (Hair et al., 2010). As the CR for Restrictive PTA was 0.736, well above the common threshold of 0.70, its convergent validity was deemed acceptable. Since supportive PTA was measured with only one indicator, its AVE and CR could not be computed, and only descriptive statistics were reported. For the remaining constructs (PI, PU, PEU, ATT, BI), their CR values were above 0.7 and AVE values were generally greater than 0.5, satisfying the criteria for convergent validity.

The Fornell–Larcker criterion confirmed discriminant validity. As shown in Table 5, for all constructs, the square root of the AVE exceeded the construct’s highest correlation with others (Fornell and Larcker, 1981). This was evidenced, for instance, by the restrictive PTA construct (sqrt (AVE) = 0.695, which was substantially larger than its maximum inter-construct correlation of 0.160 with PI, satisfying the discriminant validity requirement.

**Table 5:** Discriminant validity: Fornell–Larcker criterion.

Variables	Restrictive PTA	PI	PU	PEU	ATT	BI
Restrictive PTA	**0.695**	—	—	—	—	—
PI	0.160	**0.733**	—	—	—	—
PU	0.091	0.133	**0.723**	—	—	—
PEU	0.074	0.451	0.211	**0.783**	—	—
ATT	0.077	0.460	0.149	0.488	**0.803**	—
BI	0.107	0.470	0.082	0.404	0.511	**0.790**

Diagonal elements (in bold) represent the square root of the AVE. All the correlations were significant (*p* < 0.001). The original PTA construct was divided into single-item Supportive PTA and three-item Restrictive PTA. Due to single-indicator design, Supportive PTA was excluded from the Fornell–Larcker discriminant validity matrix; only Restrictive PTA was retained for discriminant validity test. See Table 3 for abbreviation definitions.

Theoretically, PTA was grounded in the socio-contextual view of technology acceptance and the teacher-respecting norm in Chinese culture, and thus was retained to enable a comprehensive test of social influence despite its lower AVE. Its inclusion allows for a crucial comparison with PI.

Collectively, these findings verified the sound psychometric properties of the refined measurement instrument, allowing for a rigorous empirical examination of the structural relationships and hypotheses that followed.

### 4.2 Structural model results

**Figure 2:**
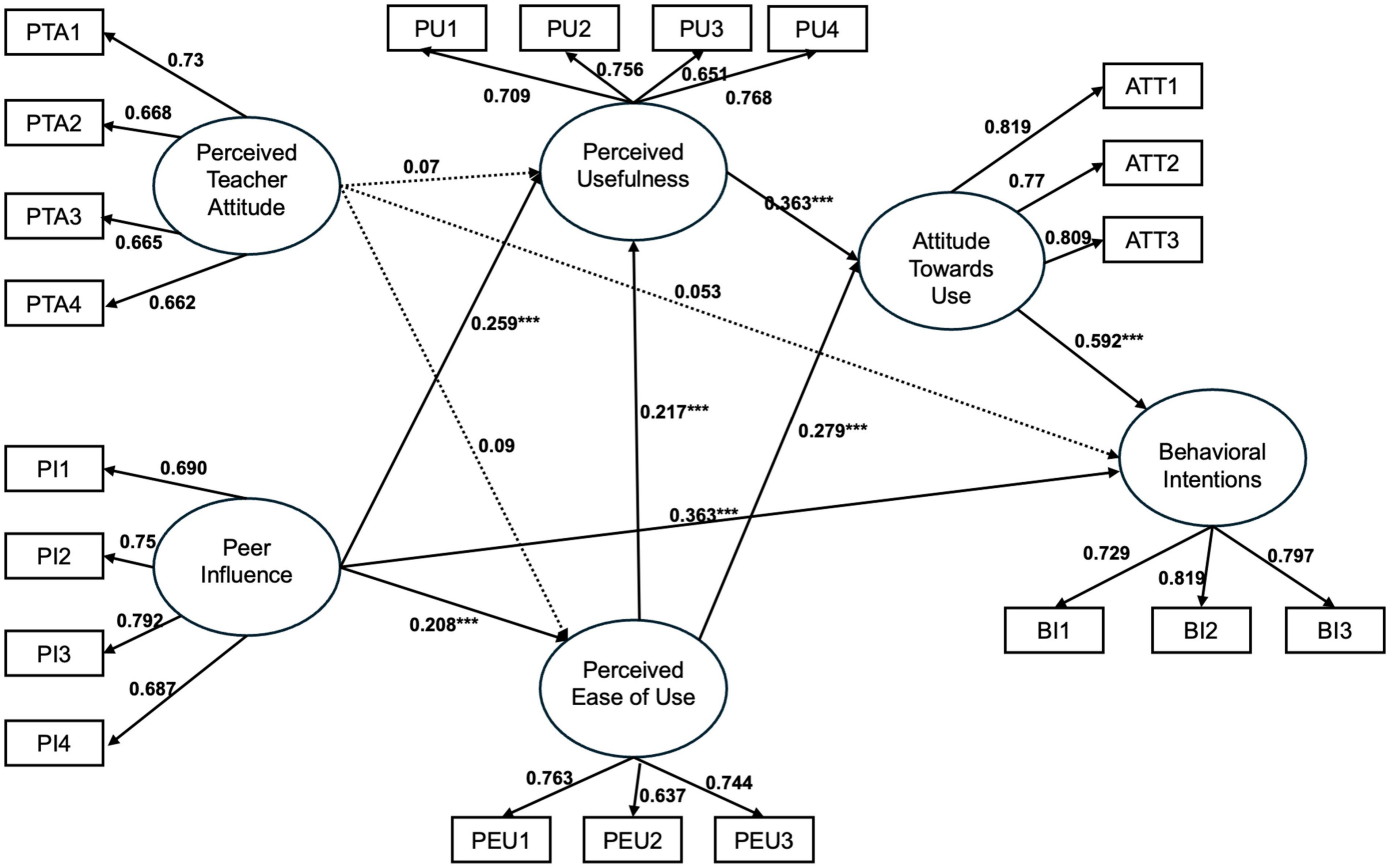
Standardized path coefficients of the final structural equation model. Asterisks denote statistical significance: *p < 0.05, **p < 0.01, ***p < 0.001. Non-significant direct paths from Perceived Teacher Attitude to Perceived Usefulness, Perceived Ease of Use, and Behavioral Intention were retained in the model for theoretical integrity.

The hypothesized structural model was assessed via SEM in RStudio (version 2025.05.01) with the lavaan and semPlot packages. Using the robust estimator, the model fit indices were as follows: *χ*^2^(177) = 431.832, *χ*^2^/df = 2.44, robust CFI = 0.919, robust TLI = 0.904, robust RMSEA = 0.062, and this model explains a significant portion of the variance in the key endogenous variables. The final model is presented in Figure 2, and the results of the hypothesis testing are summarized in Table 6.

**Table 6:** Results of the structural model.

Criterion variables	Predictor variables	Hypothesis	*β*	*t*	*p*	Results
PU	Supportive PTA	H1a	0.198	2.509	0.012	Supported
PU	Restrictive PTA	H1b	0.147	1.638	0.101	Rejected
PEU	Supportive PTA	H2a	0.080	1.190	0.234	Rejected
PEU	Restrictive PTA	H2b	0.062	0.848	0.396	Rejected
PU	PI	H3	0.259	4.477	0.000	Supported
PEU	PI	H4	0.208	3.553	0.000	Supported
PU	PEU	H5	0.217	3.845	0.000	Supported
ATT	PU	H6	0.363	6.947	0.000	Supported
ATT	PEU	H7	0.279	5.246	0.000	Supported
BI	ATT	H8	0.590	14.969	0.000	Supported
BI	Supportive PTA	H9a	0.126	2.301	0.021	Supported
BI	Restrictive PTA	H9b	−0.042	−0.672	0.502	Rejected
BI	PI	H10	0.343	7.368	0.000	Supported

H, hypothesis; others, please see Table 3 for abbreviation definitions. PTA was divided into two sub-dimensions Supportive PTA and Restrictive PTA for further analysis due to the heterogeneity of scale items.

Regarding the associations between the two dimensions of PTA and core perceptual constructs, Supportive PTA exerted a significant positive effect on PU (*β* = 0.198, *p* = 0.012), while its influence on PEU was non-significant (*β* = 0.080, *p* = 0.234). By contrast, Restrictive PTA showed no statistically significant association on either PU (*β* = 0.147, *p* = 0.101) or PEU (*β* = 0.062, *p* = 0.396). Therefore, hypotheses H2 is not supported. In contrast, PI has a significant positive structural relationship on both PU and PEU, with path coefficients of (*β* = 0.259, *p* < 0.001) and PEU (*β* = 0.208, *p* < 0.001), respectively; hypotheses H3 and H4 are both supported.

Furthermore, the classic TAM relationship between PEU and PU was confirmed, with PEU being a significant predictor of PU (*β* = 0.217, *p* < 0.001), thus supporting H5.

Regarding the determinants of ATT, both PU (*β* = 0.363, *p* < 0.001) and PEU (*β* = 0.279, *p* < 0.001) were found to be significant positive antecedents, in support of H6 and H7.

Finally, the model examined the predictors of BI. Attitude exhibited the largest relationship with BI (*β* = 0.590, *p* < 0.001). Its predictive power surpassed that of all other variables, supporting H8. In the structural model, the two dimensions of PTA showed different patterns of association with students’ behavioral intention to use Chinese GenAI tools. Supportive PTA had a marginally significant positive association with BI (*β* = 0.126, *p* = 0.021). In contrast, restrictive PTA was not significantly associated with BI (*β* = −0.042, *p* = 0.502). PI was a significant positive predictor of on BI (*β* = 0.343, *p* < 0.001), supporting H10.

After controlling for gender, familiarity with GenAI tools, and frequency of GenAI use, the structural model demonstrated that all hypothesized paths remained statistically significant. The results showed that frequency of use was significantly associated with BI (*β* = 0.188, *p* < 0.001), while gender (*β* = −0.043, *p* > 0.05) and familiarity (*β* = 0.008, p > 0.05) were not. These results indicate that the research model possesses good explanatory power and stability after accounting for potential confounding factors.

Construct level *R*^2^ values were used to evaluate the model’s ability to explain variance in endogenous constructs, a key SEM assessment metric (Hair et al., 2021). Specifically, the model explained 16.3% of the variance in PU and 5.5% of the variance in PEU. A considerably larger proportion of variance was explained in ATT, with an *R*^2^ value of 26.8%. A note on the correlation between PU and BI is warranted. The zero-order correlation between PU and BI was relatively low, yet the structural model revealed a significant indirect effect of PU on BI through ATT. This pattern is consistent with a fully mediated model, where the effect of PU on BI is transmitted entirely via ATT. As such, the low direct correlation does not invalidate the model but rather supports the mediating role of attitude in the TAM framework. Most notably, the model demonstrated strong predictive accuracy for BI, explaining 55.8% of its variance, which indicates that the included antecedents collectively form a set of predictors for students’ intention to adopt Chinese GenAI tools.

### 4.3 Mediation results

Bootstrapping analysis (5,000 samples) yielded a clear finding: PI is a powerful driver of adoption intention. By contrast, neither dimension of PTA exerted a significant indirect effect (see Table 7).

**Table 7:** Mediation path analysis results.

Path	Indirect effect	SE	95%CI
Supportive PTA → PU → ATT → BI	0.073	0.081	[−0.014, 0.304]
Supportive PTA → PEU → ATT → BI	0.023	0.019	[−0.016, 0.056]
Restrictive PTA → PU → ATT → BI	0.054	0.066	[−0.039, 0.218]
Restrictive PTA → PEU → ATT → BI	0.017	0.016	[−0.018, 0.044]
PI → PU → ATT → BI	0.016	0.008	[0.003, 0.033]
PI → PEU → PU → ATT → BI	0.003	0.002	[0.000, 0.008]
PI → PEU → ATT → BI	0.012	0.007	[0.001, 0.028]
PEU → PU → ATT → BI	0.022	0.008	[0.009, 0.039]
PEU → ATT → BI	0.086	0.019	[0.051, 0.124]

See Table 3 for abbreviation definitions. PTA was divided into two sub-dimensions Supportive PTA and Restrictive PTA for further analysis due to the heterogeneity of scale items.

PI significantly predicted intention through key sequential mediators, specifically via the sequential mediators of PU and ATT (*β* = 0.016, 95% CI [0.003, 0.033]) and PEU and ATT (*β* = 0.012, 95% CI [0.001, 0.028]). However, the longer chain mediation path through PEU, PU, and ATT was not significant (*β* = 0.003, 95% CI [0.000, 0.008]), suggesting that while PEU and PU are both important, their specific sequential combination in transmitting peer influence may be redundant in the presence of more direct routes. Overall, these significant findings indicate that students’ intention to use Chinese GenAI tools is substantially shaped by peer opinions, which inform their cognitive and affective evaluations.

Conversely, none of the hypothesized indirect paths from the two dimensions of PTA to ATT were statistically significant. Although the indirect effect of Supportive PTA via PU showed a marginally significant *p*-value (*p* = 0.073), the 95% Bootstrap confidence interval included zero ([−0.014, 0.304]), failing to support mediation. All other indirect paths were also non-significant. This suggests that for this cohort of foreign language students, the perceived attitudes of their teachers were not significantly associated with their own acceptance decisions through the proposed technology acceptance mechanisms. Finally, the core relationships of the technology acceptance model were strongly validated. PEU has a significant indirect relationship with intention via ATT (*β* = 0.086, 95% CI [0.051, 0.124]), confirming the model’s validity in this learning context.

## 5 Discussion

### 5.1 The differential relationship of external factors on core TAM constructs

To examine foreign language majors’ acceptance of Chinese GenAI tools, this study introduced two external variables: PTA and PI, aiming to investigate their relationship with foreign language majors’ behavioral intentions toward using this technology. The theoretical model was partially supported, with seven out of the 10 hypothesized paths being statistically significant. The research findings were summarized and discussed across three dimensions: external factors, TAM core constructs, and mediating variables.

Notably, the present study found that the two dimensions of PTA had different associations with foreign language majors’ BI to use Chinese GenAI tools. Supportive PTA showed a marginally significant positive association with BI, while restrictive PTA was not significantly associated with BI.

These findings are partially supported by research on student autonomy in technology adoption. Studies indicate that the current digital generation was born into a digital environment that reduces the perceived need for instructor guidance. Conversely, advice from instructors might be perceived as imposed, which could undermine its perceived benefits (Chavoshi and Hamidi, 2019; Hussein et al., 2024). Our results extend this logic by showing that restrictive teacher attitudes, those that impose boundaries or prohibitions, do not significantly deter students from adopting GenAI tools. The non-significance of restrictive PTA may be attributed to the existing linguistic abilities of foreign language majors, which likely enable them to independently evaluate the quality and practicality of GenAI outputs (Shaalan and Ahmad, 2024; Swain, 1985), thereby reducing their reliance on instructor approval or disapproval. Furthermore, the use of AI tools is often self-directed and occurs in unsupervised environments, making the process invisible to educator (Kasneci et al., 2023). Under such conditions, teacher prohibitions may simply be ignored because they cannot be enforced. This autonomy and lack of transparency directly challenge conventional methods of monitoring student progress and authenticating learning (Barrot, 2023).

The marginal significance of supportive PTA suggests that when teachers actively endorse GenAI tools rather than merely refraining from restriction, students may be more inclined to adopt them. This partial finding appears to be consistent with several studies in the existing literature (Ma, 2025; Milošević et al., 2015; Zheng et al., 2024) that demonstrate the significant role of instructors on technology adoption.

However, this non-significant result does not negate the value of teachers but rather reveals a fact that teachers’ function in the classroom has evolved alongside the advancement of digital technology. When students are confident that their teacher will offer timely support, their anxiety toward adopting new technological tools tends to diminish (Iqbal and Bhatti, 2017) This is precisely why a supportive approach that accommodates student autonomy is more conducive to integrating AI in education than attitude-controlled regulation (Ofosu-Ampong et al., 2023). As research confirms, the key to transforming student-driven AI tools from efficiency aids into enhancers of learning lies in teachers leveraging their technological knowledge to guide deep tool usage and meaningful subject integration (Kasneci et al., 2023).

In contrast to the negligible relationship involving restrictive PTA, PI emerged as a potent and multifaceted driver of both cognitive perceptions and behavioral intentions among foreign language majors.

This finding regarding peer influence aligns with the established theoretical framework of SI in the UTAUT2 model (Venkatesh et al., 2012), and is further corroborated by recent research on GenAI acceptance, which highlights students’ reliance on peer recommendations (Ma, 2025; Ofosu-Ampong et al., 2023). Given the established association between PI and adoption intention, it is imperative to translate this insight into concrete pedagogical strategies. We propose that educators formally harness this social dynamic by deliberately integrating structured peer demonstrations and collaborative problem-solving tasks into the curriculum, thereby leveraging PI to shape key learning outcomes.

### 5.2 Direct relationships among the TAM core constructs

Among foreign language majors, the PEU of Chinese GenAI tools is positively associated with PU, which confirms Hypothesis 5. This finding dovetails with the conclusions reached in other studies (Li, 2023; Liu and Ma, 2023; Wang and Wang, 2025). Notably, PEU serves as a critical predictor for evaluating and forecasting the acceptance of AI (Wang and Wang, 2025). Intuitive and user-friendly AI operations foster perceptions of usefulness, which drives greater acceptance and subsequent adoption (Li, 2023).

PU and PEU significantly predict ATT, validating Hypothesis 6 and 7. This finding corroborate earlier work (Belda-Medina and Calvo-Ferrer, 2022; Liu et al., 2024) and reinforces the established view that learners’ attitudes toward AI are primarily shaped by their functional and usability assessments (Alfadda and Mahdi, 2021). Specifically, PU fosters a positive attitude and continued usage intention by highlighting the technology’s benefits, while PEU reduces cognitive load on learners to use AI technology. For educators, this implies that they can promote student adoption by illustrating the accessibility of GenAI tools for student engagement, while also emphasizing the benefits associated with their implementation (Kasneci et al., 2023; Zheng et al., 2024).

ATT positively affect BI to use AI, supporting Hypothesis 8. This indicates that a positive attitude fosters learners’ BI to use AI (Zou et al., 2023). This general finding holds true within the particular setting of EFL learners’ informal digital English learning, where EFL learners with positive attitudes demonstrate a stronger intention to use AI technologies (Liu and Ma, 2023). This translation from attitude to intention is crucial because it suggests that fostering positive attitudes is not an end, but a powerful lever for motivating actual interaction with GenAI tools within self-directed learning settings.

### 5.3 The mediating role of perceived usefulness and perceived ease of use

This study found that PU and PEU served as significant mediators between PI and BI, with PU showing a larger indirect association than PEU. Within the Chinese foreign language learning context, characterized by a collectivist academic culture (Zhao et al., 2021), peer interactions with classmates or roommates facilitate observational learning and social validation. Students observe peers effectively utilizing GenAI tools for tasks, which not only reinforces PU through demonstrated efficacy but also enhances PEU by reducing technical apprehension through vicarious learning. These socially reinforced perceptions of utility and ease collectively foster positive attitudes toward AI adoption, thereby further enhancing adoption intention of using these tools in specialized study.

Our findings confirm a core tenet of the TAM (Davis, 1989): PU significantly shapes BI both directly and indirectly via ATT. Extending this premise to the language learning context, we find that students develop a positive attitude and subsequent willingness to use GenAI when they perceive it as effectively addressing fundamental learning tasks (Ofosu-Ampong et al., 2023), thereby transforming linguistic challenges into opportunities for proactive engagement.

Additionally, ATT plays a significant mediating role between PEU and BI, consistent with the paradigm established by Davis (1989). This means that AI tools viewed as user-friendly cultivate more optimistic attitudes, thereby enhancing behavioral intention (Wang and Wang, 2025). This entire pathway is indicative of a pragmatic, functionality-driven assessment by learners (Zou et al., 2023).

## 6 Conclusion

This study explored the adoption intention of Chinese GenAI Tools by foreign language majors, using an expanded TAM framework. The results show that PI had a significant direct relationship with foreign language majors’ BI to use Chinese GenAI translation tools, as well as indirect associations mediated through PEU and PU. Among the predictors, ATT had the largest direct relationship with BI, and PU showed a significant indirect relationship through its mediation. Regarding the two dimensions of PTA, restrictive PTA had no direct or indirect significant relationship with students’ adoption intention. In contrast, supportive PTA showed a marginally significant positive direct association.

### 6.1 Contributions of this study

This study supplements and expands the scope of application of the TAM by incorporating external variables specific to the acceptance of GenAI tools, it further subdivides the key external variable of “social influence” into two dimensions: perceived teacher attitude and peer influence, clearly distinguishing their distinct roles.

In previous studies of technology-based leaning, such as mobile learning and computer-assisted learning, teachers’ attitudes are positively associated with students’ use intention of the technology. However, this study found that AI technology challenges this conventional understanding: when foreign language majors use AI for translation and completing assignments, their acceptance is not significantly associated with their instructors’ opposed attitudes. This finding breaks through the limitations of TAM in traditional technology-assisted settings and reveals its unique applicability in scenarios where AI is integrated with foreign language instruction. This research extends the limited scholarship on AI-integrated foreign language instruction in other developing nations.

### 6.2 Practical implication

From a practical standpoint, the results suggest that policymakers, university administrators, and foreign language instructors should take actions to deal with the changes accordingly. The core challenge facing foreign language education in the future lies in the fact that the traditional exam-oriented or skills-training model is no longer sustainable; talent development must shift toward the core track of “human-AI collaboration.”

At the university level, education authorities grounded in societal development needs and workplace talent profiles, proactively anticipate the challenges facing foreign language programs in the AI era and construct a “human-AI collaboration”-oriented theoretical framework for foreign language education.

Higher education institutions should adjust their talent development programs to embed AI literacy and critical thinking within core educational objectives. At the same time, they should establish a systematic teacher training system focused on practical training in the application of GenAI in specific contexts such as translation and linguistics.

Teachers must proactively adapt to technological changes and optimize classroom instructional design. In teaching practice, the focus should be on guiding students to master the core skills of “prompt engineering,” “critical evaluation and appreciation of AI outputs,” and “post-editing.” Through a human-machine collaborative teaching model, it is possible to not only boost learners’ overall linguistic competence but also foster their core competitiveness in leveraging AI for efficiency and drawing on their professional expertise.

## 7 Limitation and future direction

Several limitations should be acknowledged. First, the cross-sectional design prevents causal inference. Future research should employ longitudinal designs to examine how students’ perceptions of teacher attitudes and peer influence evolve over time and whether they predict actual adoption behavior.

Second, the supportive dimension of PTA was measured with a single item due to the original scale structure. While single-item measures can be acceptable for concrete constructs, future research should develop additional items to capture supportive teacher attitudes more comprehensively.

Third, the sample was drawn from only two Chinese provinces (Guangxi and Hunan), which limits the generalizability of the findings to the broader population of Chinese foreign language students, future research should include institutions from more geographically diverse regions to enhance external validity.

Fourth, this study conducted EFA and CFA based on the same sample. Although all scales were mature and psychometrically sound, this approach may cause model overfitting to a certain extent. Due to the limitation of sample size, a split-sample strategy was not adopted. Future research is suggested to collect larger datasets and use independent subsamples for exploratory and confirmatory factor analysis, respectively.

Fifth, the model explained only 5.5% of the variance in PEU and 16.3% of the variance in PU. These relatively low *R*^2^ values suggest that the external variables included in this study (PTA and PI) are not sufficient to fully explain the cognitive antecedents of the TAM. Future research should incorporate additional variables such as technological self-efficacy, AI anxiety, trust, prior experience, perceived tool quality, and AI literacy to enhance the model’s explanatory power.

Finally, this study focuses on domestic Chinese GenAI platforms (DeepSeek, Doubao, ERNIE Bot). Future research could conduct systematic comparative analyses between domestic and international GenAI tools (e.g., ChatGPT) to examine how different sociotechnical environments shape learners’ technology adoption intentions.

## Data Availability

The raw data supporting the conclusions of this article will be made available by the authors, without undue reservation.

## Appendix

Core Constructs of Technology Acceptance [The items in this section are adapted from Davis (1989) and Venkatesh et al. (2003)]

Perceived usefulness

- Using Chinese generative AI tools (e.g., DeepSeek, Doubao, ERNIE Bot) helps me complete language learning tasks more quickly, such as writing and translation.
- Using Chinese generative AI tools helps me understand complex sentence structures/grammar more accurately.
- The output from Chinese generative AI tools typically requires only minor modifications before it can be used.
- I believe Chinese generative AI tools are valuable for my foreign language learning.

Perceived ease of use

- The functional layout of Chinese generative AI tools aligns with my operating habits.
- Learning to use Chinese generative AI tools does not require much effort.
- I can quickly locate the specific Chinese generative AI functions I need (e.g., document translation, speaking practice, generating contextual examples).

Attitude towards use

- Using Chinese generative AI tools makes me feel at ease.
- I am satisfied with the overall effectiveness of Chinese generative AI tools.
- I hold a positive attitude towards Chinese generative AI tools.

Behavioral intention

- I intend to continue using Chinese generative AI tools in the future.
- I am very likely to recommend that my classmates use Chinese generative AI tools.
- I will use Chinese generative AI tools in more scenarios (e.g., thesis writing, business emails).

External variables

Perceived Teacher Attitude [the PTA items are drawn from the established scale by Venkatesh et al. (2012)]

- My teachers have clearly defined the appropriate boundaries for using Chinese generative AI tools (e.g., permitting word lookups but prohibiting full-paragraph translation).
- My teachers have explicitly prohibited the use of Chinese generative AI tools in assignments.
- My teachers have acknowledged the value of Chinese generative AI tools in foreign language learning.
- My teachers criticize students who use Chinese generative AI tools in their assignments.

Peer Influence [the PI items are adapted from the classic scale by Ajzen (1991)]

- I proactively ask my classmates for specific usage tips (e.g., “How to use DeepSeek for translating academic literature”).
- I prioritize trying tools that are recommended by my classmates.
- In group assignments, I tend to use Chinese generative AI tools when other group members use them.
- My peers generally believe that using Chinese generative AI tools is beneficial.

## References

[ref1] Ajzen I. (1991). The theory of planned behavior. Organ. Behav. Hum. Decis. Process. 50, 179–211. doi: 10.1016/0749-5978(91)90020-T

[ref2] Alfadda H. A. Mahdi H. S. (2021). Measuring students’ use of zoom application in language course based on the technology acceptance model (TAM). J. Psycholinguist. Res. 50, 883–900. doi: 10.1007/s10936-020-09752-1, 33398606 PMC7781650

[ref3] Barrot J. S. (2023). Using ChatGPT for second language writing: pitfalls and potentials. Assess. Writ. 57:100745. doi: 10.1016/j.asw.2023.100745, 38826717

[ref4] Belda-Medina J. Calvo-Ferrer J. R. (2022). Using chatbots as AI conversational partners in language learning. Appl. Sci. 12:8427. doi: 10.3390/app12178427

[ref5] Chavoshi A. Hamidi H. (2019). Social, individual, technological and pedagogical factors influencing mobile learning acceptance in higher education: a case from Iran. Telematics Inform. 38, 133–165. doi: 10.1016/j.tele.2018.09.007, 38826717

[ref6] Chen Y.-c. (2022). Effects of technology-enhanced language learning on reducing EFL learners’ public speaking anxiety. Comput. Assist. Lang. Learn. 37, 789–813. doi: 10.1080/09588221.2022.2055083, 37339054

[ref7] Davis F. D. (1989). Perceived usefulness, perceived ease of use, and user acceptance of information technology. MIS Q. 13, 319–340. doi: 10.2307/249008

[ref8] Feng G. C. Su X. Lin Z. He Y. Luo N. Zhang Y. (2021). Determinants of technology acceptance: two model-based meta-analytic reviews. J. Mass Commun. Q. 98, 83–104. doi: 10.1177/1077699020952400

[ref9] Fornell C. Larcker D. F. (1981). Evaluating structural equation models with unobservable variables and measurement error. J. Mark. Res. 18, 39–50. doi: 10.1177/002224378101800104

[ref10] Granić A. Marangunić N. (2019). Technology acceptance model in educational context: a systematic literature review. Br. J. Educ. Technol. 50, 2572–2593. doi: 10.1111/bjet.12864

[ref11] Habibi A. Muhaimin M. Danibao B. K. Wibowo Y. G. Wahyuni S. Octavia A. (2023). ChatGPT in higher education learning: acceptance and use. Comput. Educ. Artif. Intell. 5:100190. doi: 10.1016/j.caeai.2023.100190

[ref12] Hair J. F. Black W. C. Babin B. J. Anderson R. E. (eds.). (2010). Multivariate Data Analysis. 7th Edn. Upper Saddle River: Prentice Hall.

[ref13] Hair J. F. Hult G. T. M. Ringle C. M. Sarstedt M. Danks N. P. Ray S. (2021). Partial Least Squares Structural Equation Modeling (PLS-SEM) Using R: A Workbook. Classroom Companion: Business. Cham: Springer International Publishing.

[ref14] Hair J. F. Jr. Sarstedt M. Hopkins L. Kuppelwieser V. G. (2014). Partial least squares structural equation modeling (PLS-SEM): an emerging tool in business research. Eur. Bus. Rev. 26, 106–121. doi: 10.1108/EBR-10-2013-0128

[ref15] Hatamleh H. A. Alsaadi O. Alkhafaji B. Khasawneh M. A. S. Tashtoush M. A. (2025). Game-based and AI-driven engagement strategies to combat demotivation in foreign language learning. Int. J. Adv. Appl. Sci. 12, 119–130. doi: 10.21833/ijaas.2025.03.013

[ref16] Hayes A. F. (2013). Introduction to Mediation, Moderation, and Conditional Process Analysis: A Regression-Based Approach. Methodology in the Social Sciences. New York: The Guilford Press.

[ref17] Hsu L. (2022). EFL learners’ self-determination and acceptance of LMOOCs: the UTAUT model. Comput. Assist. Lang. Learn. 36, 1177–1205. doi: 10.1080/09588221.2021.1976210

[ref18] Hu L.-t. Bentler P. M. (1999). Cutoff criteria for fit indexes in covariance structure analysis: conventional criteria versus new alternatives. Struct. Equ. Model. 6, 1–55. doi: 10.1080/10705519909540118

[ref19] Hussein L. A. Alqarni K. Hilmi M. F. Agina M. F. Shirawia N. Abdelreheem K. I. . (2024). The mediating role of learning management system use in enhancing system effectiveness. WSEAS Trans. Bus. Econ. 21, 2067–2078. doi: 10.37394/23207.2024.21.169, 42157329

[ref20] Iqbal S. Bhatti Z. A. (2017). What drives M-learning? An empirical investigation of university student perceptions in Pakistan. High. Educ. Res. Dev. 36, 730–746. doi: 10.1080/07294360.2016.1236782

[ref21] Kasneci E. Sessler K. Küchemann S. Bannert M. Dementieva D. Fischer F. . (2023). ChatGPT for good? On opportunities and challenges of large language models for education. Learn. Individ. Differ. 103:102274. doi: 10.1016/j.lindif.2023.102274, 38826717

[ref22] Kline R. (2015). Principles and Practice of Structural Equation Modeling. 4th. Methodology in the Social Sciences. New York: The Guilford Press.

[ref23] Kock N. Moqbel M. (2016). A six-stage framework for evolutionary IS research using path models: conceptual development and a social networking illustration. J. Syst. Inf. Technol. 18, 64–88. doi: 10.1108/JSIT-04-2015-0028

[ref24] Kohnke L. Moorhouse B. L. Zou D. (2023). ChatGPT for language teaching and learning. RELC J. 54, 537–550. doi: 10.1177/00336882231162868

[ref25] Lai Y. L. Lee J. (2020). Integration of technology readiness index (TRI) into the technology acceptance model (TAM) for explaining behavior in adoption of BIM. Asian Educ. Stud. 5:10. doi: 10.20849/aes.v5i2.816

[ref26] Li K. (2023). Determinants of college students’ actual use of AI-based systems: an extension of the technology acceptance model. Sustainability 15:5221. doi: 10.3390/su15065221

[ref27] Liang J.-C. Hwang G.-J. Chen M.-R. A. Darmawansah D. (2021). Roles and research foci of artificial intelligence in language education: an integrated bibliographic analysis and systematic review approach. Interact. Learn. Environ. 31, 4270–4296. doi: 10.1080/10494820.2021.1958348, 37339054

[ref28] Lin J. Li J. (2025) Personalized learning in college English education: leveraging large language models for adaptive instruction 2025 2nd international conference on informatics education and computer technology applications (IECA) 119–125

[ref29] Liu G. L. Darvin R. Ma C. (2024). Exploring AI-mediated informal digital learning of English (AI-IDLE): a mixed-method investigation of Chinese EFL learners’ AI adoption and experiences. Comput. Assist. Lang. Learn. 38, 1632–1660. doi: 10.1080/09588221.2024.2310288

[ref30] Liu G. Ma C. (2023). Measuring EFL learners’ use of ChatGPT in informal digital learning of English based on the technology acceptance model. Innov. Lang. Learn. Teach. 18, 125–138. doi: 10.1080/17501229.2023.2240316, 37339054

[ref31] Ma M. (2025). Exploring the acceptance of generative artificial intelligence for language learning among EFL postgraduate students: an extended TAM approach. Int. J. Appl. Linguist. 35, 91–108. doi: 10.1111/ijal.12603

[ref32] Meyer J. Jansen T. Schiller R. Liebenow L. W. Steinbach M. Horbach A. . (2024). Using LLMs to bring evidence-based feedback into the classroom: AI-generated feedback increases secondary students’ text revision, motivation, and positive emotions. Comput. Educ. Artif. Intell. 6:100199. doi: 10.1016/j.caeai.2023.100199, 38826717

[ref33] Milošević I. Živković D. Manasijević D. Nikolić D. (2015). The effects of the intended behavior of students in the use of M-learning. Comput. Hum. Behav. 51, 207–215. doi: 10.1016/j.chb.2015.04.041, 38826717

[ref34] Moradi H. (2025). Integrating AI in higher education: factors influencing ChatGPT acceptance among Chinese university EFL students. Int. J. Educ. Technol. High. Educ. 22:30. doi: 10.1186/s41239-025-00530-4

[ref35] Moussalli S. Cardoso W. (2020). Intelligent personal assistants: can they understand and be understood by accented L2 learners? Comput. Assist. Lang. Learn. 33, 865–890. doi: 10.1080/09588221.2019.1595664

[ref36] Nunnally J. C. Bernstein I. H. (1994). Psychometric Theory. 3rd Edn. New York: McGraw-Hill.

[ref37] Ofosu-Ampong K. Acheampong B. Kevor M. O. Amankwah-Sarfo F. (2023). Acceptance of artificial intelligence (ChatGPT) in education: trust, innovativeness and psychological need of students. Inf. Knowl. Manag. 13, 37–47. doi: 10.7176/IKM/13-4-03

[ref38] Panopoulou E. Tambouris E. Tarabanis K. (2021). An eParticipation acceptance model. IEEE Trans. Emerg. Top. Comput. 9, 188–199. doi: 10.1109/TETC.2018.2861426

[ref39] Podsakoff P. M. MacKenzie S. B. Lee J.-Y. Podsakoff N. P. (2003). Common method biases in behavioral research: a critical review of the literature and recommended remedies. J. Appl. Psychol. 88, 879–903. doi: 10.1037/0021-9010.88.5.879, 14516251

[ref40] Shaalan I. E.-N. A. W. Ahmad A. S. K. (2024). Linguistic competence among Egyptian vs. Saudi EFL majors in light of utilizing artificial intelligence technology: a predictive study. Int. J. Comput. Assist. Lang. Learn. Teach. 14, 1–19. doi: 10.4018/IJCALLT.361771

[ref41] Swain M. (1985). Input in second language acquisition. Mod. Lang. J. 70:172. doi: 10.2307/327330

[ref42] Venkatesh Morris Davis Davis (2003). User acceptance of information technology: toward a unified view. MIS Q. 27:425. doi: 10.2307/30036540

[ref43] Venkatesh V. Thong J. Y. L. Xu X. (2012). Consumer acceptance and use of information technology: extending the unified theory of acceptance and use of technology. MIS Q. 36, 157–178. doi: 10.2307/41410412

[ref44] Wang W. Wang W. (2025). College students’ behavioural intentions of AI-assisted language learning: based on the technology acceptance model. J. Comput. Assist. Learn. 41:e70075. doi: 10.1111/jcal.70075

[ref45] Wu X. Zhang Y. (2024). Research on the application of ERNIE bot in college English teaching. Int. J. Educ. Humanit. 12, 216–219. doi: 10.54097/ynbb1n08

[ref47] Zhang Q. (2024). The affective and performative effects of ludicization on language learning: an integrated model related to technology acceptance and multidimensional motivation. Educ. Inf. Technol. 29, 17293–17326. doi: 10.1007/s10639-024-12538-w

[ref48] Zhang Y. Yu Z. (2025). Emotional attachment as the key mediator: expanding UTAUT2 to examine how perceived anthropomorphism in intelligent agents influences the sustained use of DouBao (Cici) among EFL learners. Educ. Inf. Technol. 30, 23897–23919. doi: 10.1007/s10639-025-13721-3

[ref49] Zhao S. Feng J. Wang P. Ma L. Wen J. (2025). Empowering college English teaching with Deepseek: practical applications and challenges. Sino-US Engl. Teach. 22, 153–157. doi: 10.17265/1539-8072/2025.05.001

[ref50] Zhao Y. Wang N. Li Y. Zhou R. Li S. (2021). Do cultural differences affect users’ e-learning adoption? A meta-analysis. Br. J. Educ. Technol. 52, 20–41. doi: 10.1111/bjet.13002

[ref51] Zheng Y. Wang Y. Liu K. S.-X. Jiang M. Y.-C. (2024). Examining the moderating effect of motivation on technology acceptance of generative AI for English as a foreign language learning. Educ. Inf. Technol. 29, 23547–23575. doi: 10.1007/s10639-024-12763-3

[ref52] Zou B. Lyu Q. Han Y. Li Z. Zhang W. (2023). Exploring students’ acceptance of an artificial intelligence speech evaluation program for EFL speaking practice: an application of the integrated model of technology acceptance. Comput. Assist. Lang. Learn. 38, 1366–1391. doi: 10.1080/09588221.2023.2278608

